# Spectral Behavior of a Conjugated Polymer MDMO-PPV Doped with ZnO Nanoparticles: Thin Films

**DOI:** 10.3390/nano13172405

**Published:** 2023-08-24

**Authors:** Boutheina Ben Abdelaziz, Nazir Mustapha, Idriss M. Bedja, Osamah Aldaghri, Hajo Idriss, Moez Ibrahem, Khalid H. Ibnaouf

**Affiliations:** 1 Advanced Materials and Quantum Phenomena Laboratory, Physics Department, Faculty of Sciences of Tunis, Tunis El-Manar University, 2092 University Campus, Tunis 1006, Tunisia; bouthainabenabdelaziz@gmail.com; 2 Physics Department, College of Science, Imam Mohammad Ibn Saud Islamic University (IMSIU), Riyadh 13318, Saudi Arabia; nmmustapha@imamu.edu.sa (N.M.); hiidriss@imamu.edu.sa (H.I.); maimohammed@imamu.edu.sa (M.I.); 3 Cornea Research Chair, Department of Optometry, College of Applied Medical Sciences, King Saud University, P.O. Box 10219, Riyadh 11433, Saudi Arabia; bedja@ksu.edu.sa

**Keywords:** MDMO-PPV, ZnO nanoparticles, thin film, photoluminescence spectroscopy, Franck Condon

## Abstract

The purpose of the presented study is to examine the impact of zinc oxide nanoparticles (ZnO NPs) on the spectrum features of poly [2-methoxy-5-(3′,7′-dimethyloctyloxy)-1, 4-phenylenevinylene] (MDMO-PPV). The characteristics of the MDMO-PPV and doped ZnO NPS samples were assessed using several techniques. A set of solutions of MDMO-PPV in toluene that were doped with different ratio percentages of ZnO NPs was prepared to obtain thin films. Pristine and composite solutions were spin-coated on glass substrates. It was observed that MDMO-PPV had two distinct absorbance bands at 310 and 500 nm in its absorption spectrum. The UV-Vis spectrum was dramatically changed when 5% of ZnO NPs were added. The result showed a significant reduction in absorption of the band 500 nm, while 310 nm absorption increased rapidly and became more pronounced. Upon adding (10%) ZnONPs to the sample, no noticeable change was observed in the 500 nm band. However, the 310 nm band shifted towards the blue region. There is a dominant peak in the PL spectrum of MDMO-PPV in its pristine form around 575 nm and a smaller hump around 600 nm of the spectrum. The spectral profile at 600 nm and the intensity of both bands are improved by raising the ZnO NP concentration. These bands feature two vibronic transitions identified as (0-0) and (0-1). When the dopant concentration increased to the maximum dopant percentage (10%), the energy band gap values increased by 0.21 eV compared to the pristine MDMO-PPV. In addition, the refractive index (*n*) decreased to its lowest value of 2.30 with the presence of concentrations of ZnO NPs.

## 1. Introduction

Nanomaterials and polymer composites have unique optical and electrical properties; hence, they were used in various fields of technology, including military, ceramic, fiber water treatment, targeted drug delivery, energy storage, agriculture, and engineering systems [1,2,3,4,5]. Conjugated polymers (CPs) represent an emerging category of materials that integrate the optical and electrical characteristics of semiconductors with the processing benefits and mechanical attributes of plastics [6,7]. CPs have emerged as promising materials in optoelectronic devices [8,9,10,11]. Conjugated polymers are desirable owing to various benefits, like low-cost fabrication, ease of processing, and excellent surface morphology [12]. Electronic and optoelectronic devices are fabricated using spin coatings in which conjugated polymers are deposited into a thin film of uniform thickness [13]. Studies have focused on CPs and devices to achieve the best photoluminescence (PL) and absorption characteristics in the ultraviolet and visible ranges [14,15,16]. Poly (p-phenylene vinylene) (PPV) is the most attractive material for optical and electrical applications due to their physiochemical features. [17,18]. Since 1990, PPV and its related compounds have been subjected to intense research as pioneering substances for organic optical devices [19,20,21,22,23,24,25]. Among its properties, poly [2-methoxy-5-(3′,7′-dimethyloctyloxy)-1,4-phenylenevinylene] (MDMO-PPV) is particularly suitable for device fabrication having an energy gap of 2.15 eV, MDMO-PPV is a highly prevalent conducting polymer that finds extensive utilization in the field of plastic electronics [26,27]. ZnO NPs possess high optoelectronic features, which makes them well-suited as engineering materials [28,29,30]. The utilization of nanoparticles in developing CPs has garnered significant attention in research due to its promising optical and electrical capabilities, which lead to stable and high-performance devices. [31,32,33]. The addition of ZnO NPs into PMMA polymer results in a decreased optical band gap. This decrease in the band gap is owing to the increase of the disorder in the nanocomposite generated by the presence of the localized states where many transitions into the band gap are allowed by the increase of the localized state in the nanocomposite [33]. Composites of ZnO NPs and CPs are fascinating for use in OLEDs [34,35,36,37].

This work aims to investigate the optical, structural, and morphological properties of pure MDMO-PPV thin film and its blends by incorporating ZnO NPs in two quantities, 5% and 10% and applying Franck-Condon fit, to reproduce the PL spectra.

## 2. Experimental Procedures

### 2.1. Materials Preparation

**Figure 1:**
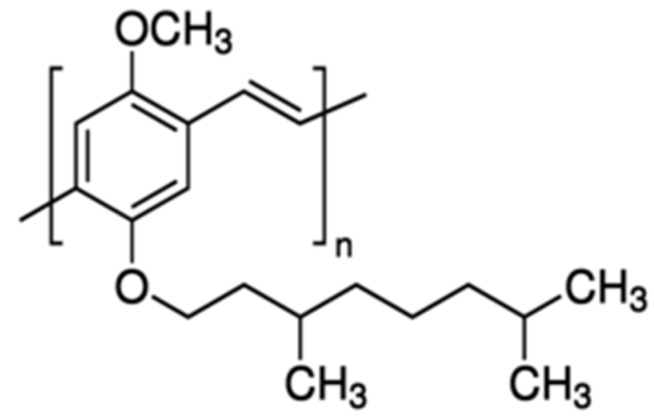
Chemical structure of MDMO-PPV.

Poly [2-methoxy-5-(3′,7′-dimethyloctyloxy)-1,4-phenylenevinylene] (MDMO-PPV) with a molecular weight of 120,000 g/mol was acquired from Sigma-Aldrich Company (Burlington, MA, USA) and used as received. Figure 1 illustrates the Chemical structure of MDMO-PPV. Solutions with various concentrations of MDMO-PPV were dissolved in toluene. Then pristine and blended polymeric solutions with 5% and 10% ZnO nanoparticles were prepared. The solutions of MDMO-PPV: ZnO NPs were mixed and agitated using a magnetic stirrer for one hour to obtain homogenous solutions. Afterwards, the solutions were deposited onto glass substrates (1×1 inch) via spin-coating (800 rpm), and then films were dried at 120 °C inside an oven for 20 min to avoid photodegradation. The thickness of the films was measured by a Deck Tack 150 and recorded in Table 1.

### 2.2. Materials Characterization

The optical properties at room temperature, specifically the absorption characteristics, of both pristine MDMO-PPV and two different blended films were assessed within the visible light wavelength range (190–900 nm) with a Perkin Elmer lambda 40 UV-Vis spectrophotometer (Waltham, MA, USA). Also, the PL spectra of these samples were measured using the Perkin Elmer LS 45 Luminescence instrument (Waltham, MA, USA). The polymeric films’ morphological features were assessed by the utilization of a JSM-7600F scanning electron microscopy (SEM) of type JEOL, Tokyo, Japan. Also, the X-ray diffraction (XRD) was performed using the Bruker-AXS D8 ADVANCE instrument (Billerica, MA, USA), utilizing Cu-kα radiation with a wavelength (λ) of 0.1542 nm. The diffraction measurements were conducted between the 2θ range of 10^0^–80^0^. The standard values for the high voltage and current were recorded as 45 (kV) and 30 (mA), respectively.

## 3. Results and Discussion

### 3.1. Optical Properties of ZnO NPs Doped MDMO-PPV

#### 3.1.1. Absorption Spectra

**Figure 2:**
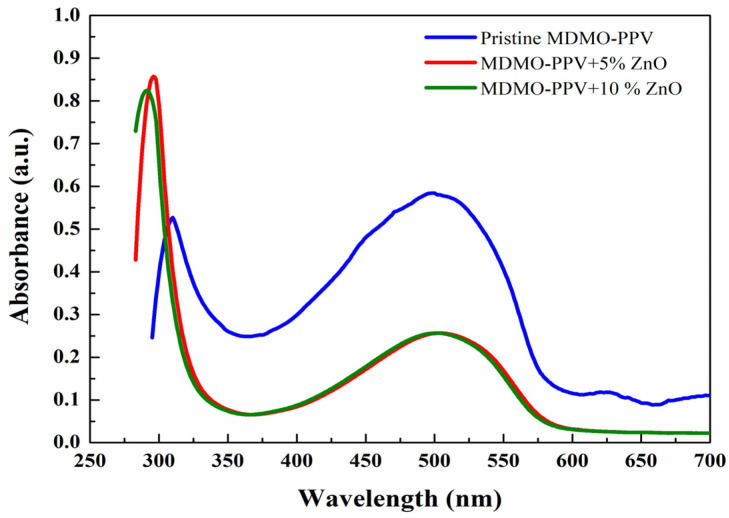
Absorbance spectra of the pristine and MDMO-PPV: ZnO NPs blends (5% and 10%) thin films.

The intramolecular charge transfer (ICT) via the π system is an important feature of some conducting polymers. The absorption spectrum showed two distinctive bands at 310 and 500 nm, as shown in Figure 2. The first absorption band (310 nm) arises from the π-π* transition within the benzenoid segments. The second absorption band at 500 nm is attributed to the doping level of polymer MDMO-PPV (polaron-π* transition) and the formation of localized polaron at the backbone of the polymer (π-polaron transition) [38]. For the doped samples, the wavelength of 500 nm  decreased its absorbance without any change in the wavelength position.

In contrast, the shorter wavelength of 310 nm became dominant, and there was an increase in its absorbance and a considerable shift towards the blue region. The observed shift toward the blue region in the absorption bands may be due to adding ZnO nanoparticles to the polymer and their interaction with MDMO-PPV [39]. When the doping ratio increased to 10%, there was no significant change in wavelength position and the absorbance for the wavelength 500 nm. In contrast, a slight change occurred in the absorbance and wavelength for the shorter wavelength of 300 nm. The findings agree with previous studies of the same compound [21].

#### 3.1.2. Energy Band Gap ($E_{g}$) and Refractive Index (n)

**Figure 3:**
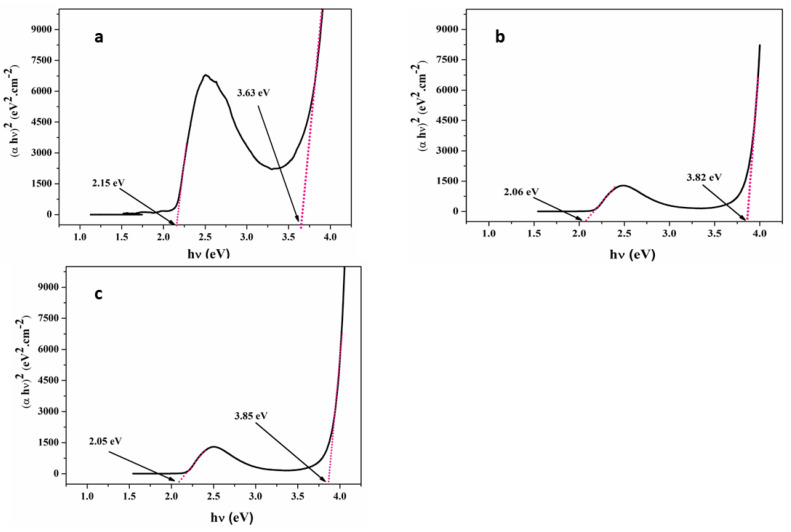
${\left(\alpha hv\right)}^{2}$ versus photon energy of (**a**) pristine and doped (**b**) MDMO-PPV: ZnO NPs (5%) (**c**) MDMO-PPV: ZnO NPs (10%) thin films.

In accordance with the recorded absorption spectra of pristine and ZnO NPs doped conducting polymer of MDMO-PPV, Using the Tauc method, the optical energy band gaps were computed as follows [40,41]:

(1) $${\left(ahv\right)}^{2}=A\left(hv-E_{g}\right)$$

where a is the absorbance coefficient, hv is the photon energy, A is the materials’ characteristic constant, and $E_{g}$ is the optical energy band gap between the valence and conduction bands. The proposed model maintains a parabolic dependance of the photon energy with variation in the absorption band’s edge. Figure 3 shows the linear relationship between ${\left(\alpha hv\right)}^{2}$ versus $\left(hv\right)$, which implies a direct band gap of the electronic transitions. The hv-axis $E_{g}$ values were determined using straight-line extrapolation, which is listed in Table 2. Figure 3a shows that MDMO-PPV thin film has two $E_{g}$ values of 2.15 eV and 3.63 eV, respectively, agree with other researchers [42]. For the dopant concentration of 5% of ZnO NPs, the low energy band gap was reduced from 2.15 eV, for pristine MDMO-PPV to 2.06. In contrast, the high band gap energy drastically increases by 0.2 eV (Figure 3b). In the case of a dopant concentration rate of 10%, the energy band gaps mentioned in the pristine case are slightly affected and shifted to 2.05 eV and 3.85 eV, respectively as displayed in Figure 3c. The decrease of this absorption band with adding ZnO NPs is related to the increase in film thickness [43].

The variation in the optical band gap is associated with changes in the density of states that are localized inside the band gap caused by unsaturated defects present in the nanocomposite or the energy shift between the valence and the conduction band. In addition, the enhancement of carrier–carrier interaction due to the high concentration of carriers in valence and conduction bands leads to a reduction in the bandgap [44]. Moreover, increasing ZnO NPs led to decreasing the formation defects in MDMO-PPV: ZnO NPs blends where electrons will fill up these defects at MDMO-PPV: ZnO NPs interface, then less localized states are formed. This effect will change the degree of disorder in the film and then the optical band gap. The Urbach relation was used to determine the width of localized states in the low absorption [45,46]:

(2) $$\alpha =\alpha_{0}exp\left(\frac{h\nu }{E_{u}}\right)$$

where α denotes the experimentally deduced optical absorption profile, the width of the tails of localized states in the energy band gap represented by $E_{u}$ (the Urbach energy interpreted), and $\alpha_{0}$ is a constant [47,48]. Equation (2) can be rearranged as:

(3) $$\ln\left(\alpha \right)=\frac{h\nu }{E_{u}}+\ln\left(\alpha_{0}\right)$$

**Figure 4:**
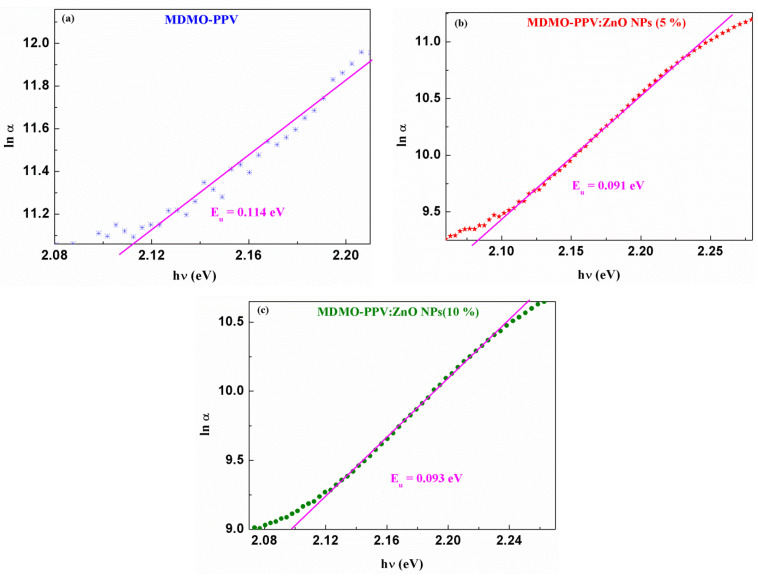
$\ln\left(\alpha \right)$ versus photon energy of (**a**) pristine and doped (**b**) MDMO-PPV: ZnO NPs (5%) (**c**) MDMO-PPV: ZnO NPs (10%) thin films.

The $\ln\left(\alpha \right)$ versus hν plots for all thin films are represented in Figure 4. Obtaining the slope gives the Urbach energy according to [49,50]:

(4) $$\frac{1}{E_{u}}=\frac{d\left(\ln\left(\alpha \right)\right)}{d\left(h\nu \right)}$$

$E_{u}\;$ values of these samples were determined and listed in Table 2. Incorporating ZnO NPs into MDMO-PPV resulted in a decrease of $E_{u}\;$ compared with the pristine polymer. These results confirm the reduction of defects and localized states in the forbidden energy gap of the blend samples. Furthermore, the $E_{u}\;$ the second blend is slightly higher than the first, which follows the band gap increase for 10% of ZnO NPs. More localized states are present in the MDMO-PPV: ZnO NPs (10%) thin film.

The refractive indices of these films were calculated via the Moss equation [51].

(5) $$n^{4}=\frac{k}{E_{g}}\;$$

where *k* is constantly equal to 108 eV. These results demonstrate that the refractive index decreases in the presence of ZnO until it reaches its minimum value of 2.30, as shown in Table 2.

#### 3.1.3. Photoluminescence Spectra

**Figure 5:**
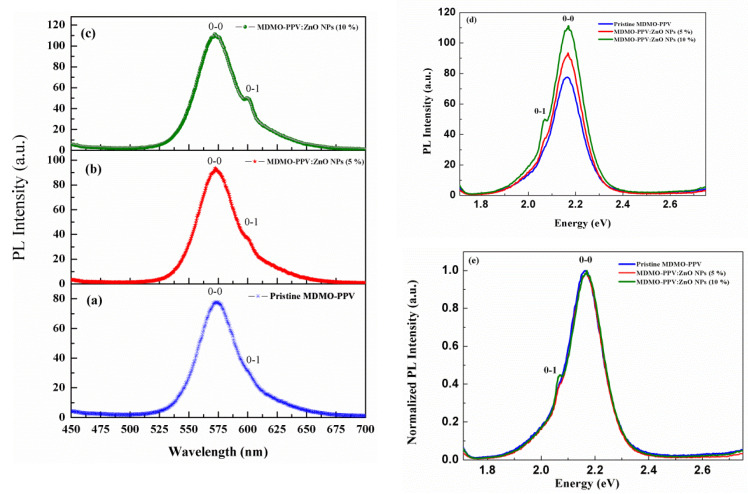
PL spectra of the (**a**) MDMO-PPV (**b**) MDMO-PPV: ZnO NPs 5% (**c**) MDMO-PPV: ZnO NPs 10%. (**d**) Non-Normalized (**e**) Normalized PL intensity of MDMO-PPV and its blends thin films.

The MDMO-PPV thin film PL spectrum shows two emission bands centered at ~575 nm and ~600 nm as illustrated in Figure 5a. These peaks are associated with the 0-0 and 0-1 vibronic transitions, respectively [52,53]. MDMO-PPV polymer emission occurs through electronic transitions between electronic states in different vibrational states [54]. The observed splitting corresponds to the energy of the out-of-plane C-H stretching vibration of the vinylene group ($\sim 963{\;\mathrm{cm}}^{-1}$) [52]. The effect of the nanoparticle concentration of impurities in ZnO NPs on the PL spectrum of MDMO-PPV: ZnO NPs in thin films was studied. As shown in Figure 5a, the PL intensity ratio between 570 nm (0-0) and 600 nm (0-1) for MDMO-PPV in its pristine form is 2.81. This PL intensity ratio is larger than 1, which suggests that an intrachain singlet exciton is the origin of this luminescence [55].

Moreover, it might be stated that in MDMO-PPV thin film, the intrachain-aggregation effect is predominant, resulting in J-aggregate-dominant. When 5% ZnO NPs added, the intensity of the ratio became 2.55, as shown in Figure 5a; besides that, no shift is observed in the wavelength position. Meanwhile, the intensity increased in the order of magnitude of 1.22, and the band at 600 nm grew slightly. For a dopant percentage of 10%, the band of 600 nm increased and became more pronounced, and the intensity ratio became 2.26 (Figure 5c). According to our present results, the small size of ZnO NPs plays a major role in enhancing PL emission in thin films. This is shown in our present findings, in which the PL intensity is increased for both blends for different concentrations of ZnO NPs.

Moreover, the trapped electrons in the defect states of ZnO NPs will be transferred to the lowest unoccupied molecular orbital (LUMO) of the MDMO-PPV polymer. These charges transferred from inorganic to organic materials enhance the PL emission. Consequently, holes at the highest occupied molecular orbital (HOMO) of the MDMO-PPV will radiatively recombine with transferred electrons to emit photons. Thus, increasing the amount of ZnO NPs in the pristine polymer leads to an increase in the exciton formation and then increases the PL emission observed at 10% of ZnO NPs. This result suggests that the MDMO-PPV: ZnO NPs (10%) blend is suitable for luminescence applications. In addition, the confinement effect of ZnO NPs over MDMO-PPV molecules can enhance PL emission. ZnO NPs protect the polymer and immobilize it within their confined space [56,57]. This metal oxide will serve as a shield to suppress the interaction among the MDMO-PPV molecules, reducing self-quenching in the excited state. The decrease in emission intensity seen with the adding more ZnO NPs can be attributed to the increased aggregation. This leads to a higher number of electrons largely localized at ZnO’s surface. This localization restricts the population of recombination events for the MDMO-PPV/ZnO [58].

Conversely, modifications to the conjugation length led to blueshifts in the PL spectra. Adding ZnO NPs to MDMO-PPV causes the polymer chains to separate, resulting in the PL spectra having a blue shift [59]. Avoiding the photo-excited state’s internal conversion and intersystem crossing mechanisms originates from the increased PL emission observed in MDMO-PPV: ZnO NPs blends [42,60]. The findings of this study are consistent with prior research [57].

### 3.2. X-ray Diffraction (XRD) Measurements

**Figure 6:**
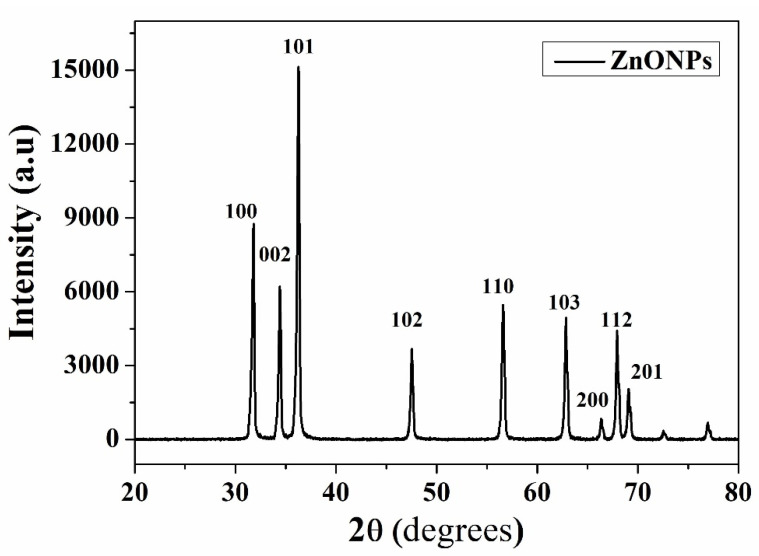
The XRD pattern obtained for ZnO NPs.

In Figure 6, the XRD patterns of pristine ZnO NPs demonstrate the characteristic peaks of the hexagonal Wurtzite Zinc Oxide structure. These peaks were observed at the diffraction angles (2θ) of 31.7, 34.4, 36.3, 47.5, 56.6, 62.8, 66.4, 67.9, and 69.1°, which correspond to the first-order and second-order lattice planes of (100), (002), (101), (102), (110), (103), (200), (112), and (201), respectively. This result confirms the hexagonal structure of the polycrystalline formation. The presence of distinct diffraction peaks suggests that ZnO nanoparticles exhibit high crystallinity. The average particle size is determined using the Scherrer formula [31]:

(6) $$D=\frac{k\;\lambda }{\beta \;cos\left(\theta \right)}$$

where D is the crystallite size, k is the shape factor correction (0.94), β  is the full widths at half maxima (FWHM) of the broadened diffracted peaks in radian (0.5°) and λ is 0.1542 nm. Calculations were performed to determine the average crystallite size of ZnO NPs is 17.3 nm.

**Figure 7:**
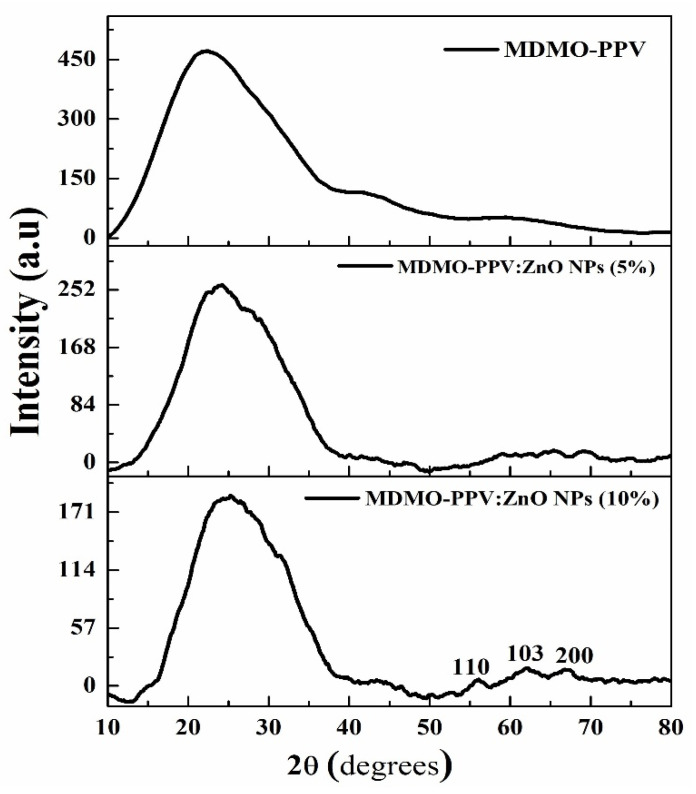
XRD pattern of pristine MDMO-PPV and MDMO-PPV: ZnO NPs.

Figure 7 depicts the X-ray diffraction of pristine and doped MDMO-PPV with two ZnO NPs ratios. The patterns exhibited that MDMO-PPV had a broad peak in 2θ from 10 to −40°, which confirms the amorphous phase. For 5% of ZnO NPs, the intensity decreased rapidly and did not show any significant difference from that of the pristine MDMO-PPV; thus, the XRD pattern profile did not change. When the ZnO NPs concentration increased to 10%, the intensity continued to decrease. Moreover, the crystallinity was slightly enhanced at lattice planes of (110), (103), and (200). This decrease in the polymer peak is related to the distribution of ZnO NPs in the MDMO-PPV matrix, where additional metal oxide inside the MDMO-PPV chain orients crystallizes the polymer chain at proper sites [61]. The appearance of the ZnO NPs diffraction peaks with increasing its concentration to MDMO-PPV is emphasizing the formation of MDMO-PPV: ZnO NPs blends. The XRD findings are in accordance with the enhancement of PL intensity with increasing ZnO NPs to 10% due to the caging effect related to the presence of this metal oxide in the blend, as shown in Figure 7.

### 3.3. Scanning Electron Microscope (SEM)

**Figure 8:**
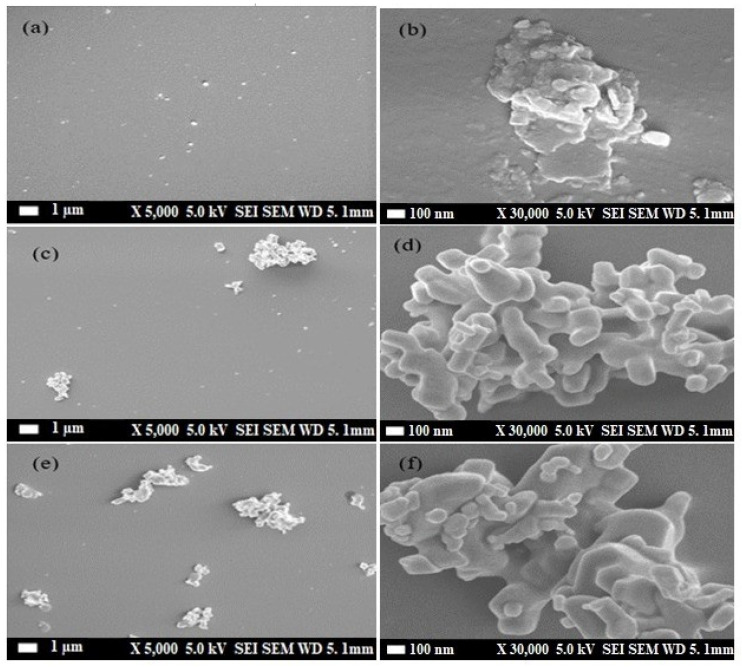
SEM visuals of (**a**,**b**) MDMO-PPV thin films (**c**,**d**) MDMO-PPV: ZnO NPs (5%) and (**e**,**f**) MDMO-PPV: ZnO NPs (10%) blended thin films, for 1 μm and 100 nm scales.

Figure 8a–f show SEM images of MDMO-PPV thin films, MDMO-PPV containing 5% of ZnO NPs, and 10% of ZnO NPs thin films, respectively at 1 μm and 100 nm scales. It’s clearly shown in Figure 8a that the pristine polymer thin film contains a small aggregate. Furthermore, the aggregate size and distribution increase with increasing ZnO NPs concentration. At higher concentrations (Figure 8e,f). The aggregation is seen in the high ZnO NP content and the non-uniform dispersion of nanoparticles on the thin film surface morphology. This aggregate, which also contains ZnO NPs in the MDMO-PPV, is essential for enhancing conformational disorder [62].

### 3.4. Theoretical Analysis of the PL Spectra

The experimental PL spectra of the MDMO-PPV polymer, shown in Figure 5d (blue spectrum), can be modeled using Franck-Condon (FC) analysis as the sum of FC transitions based on several intramolecular vibrational modes [63]. The intensity $I_{0\to \nu_{i}}$ of the vibronic transitions $0\to \nu_{i}$ for the *i* mode is given by [64]:

(7) $$I_{0\to \nu_{i}}\propto \;{\left(\hbar \omega \right)}^{3}\;n_{f}^{3}S_{i}^{\nu_{i}}\;\frac{e^{-S_{i}}}{\nu_{i}!}$$

where, $\nu_{i}$ is the vibrational level, $n_{f}$ is the refractive index real part at photon energy ℏω, $S_{i}$ is the Huang Rhys (HR) factor.

From Equation (7) we can deduce:

(8) $$n_{f}^{3}=\frac{I_{0-\nu_{i}}\;\;\nu_{i}!}{{\left(\hbar \omega \right)}^{3}\;\;S_{i}^{\nu_{i}}\;\;e^{-S_{i}}}\;$$

The $S_{i}$ factors account for the $0-\nu_{i}$ vibrational transitions where $\nu_{i}$ is attributed to the vibrational level, we use two vibrational levels (0-0 and 0-1) for the same vibrational mode ($\;\hbar \omega_{i}=\hbar \omega$ = 0.11 eV).

Thus, real part of the refractive index $n_{f0-0}$ and $n_{f0-1}$ for 0-0 and 0-1 vibrational transitions, respectively, as mentioned below were calculated as:

(9) $$n_{f0-0}=\sqrt[{3}]{\left(\frac{I_{0-0}\;0!}{{\left(\hbar \omega \right)}^{3}\;\;S_{0}^{0}\;\;e^{-S_{0}}}\right)}$$

(10) $$n_{f0-1}=\sqrt[{3}]{\left(\frac{I_{0-1}\;1!}{{\left(\hbar \omega \right)}^{3}\;\;S_{1}^{1}\;\;e^{-S_{1}}}\right)}$$

where $I_{0-0}$ and $I_{0-1}$ are the maximum of the PL intensity for the transitions 0-0 and 0-1 respectively

Furthermore, the $S_{i}$ factor represents the average number of phonons engaged in the emission process. Which can be written as: [65]

(11) $$S_{i}=\frac{1}{2}\frac{M_{i}\;\;\omega_{i}^{2}}{\hbar \omega_{i}}{\left(\Delta Q_{i}\right)}^{2}$$

where, $\Delta Q_{i}$ is the displacement between the ground and excited states, $M_{i}$ is the reduced ionic mass for the *i* mode. This HR factor is also associated with the relaxation energy $E_{rel}$, which serves as an indicator of the electron-phonon coupling’s intensity [66]:

(12) $$E_{rel}=\sum_{i}S_{i}\hbar \omega_{i}$$

The displacement between the ground and excited states ${\Delta Q}_{0}$ and ${\Delta Q}_{1}$ of the 0-0 and 0-1 transitions respectively can be calculated using: [58]

(13) $$\Delta Q_{0}{}^{2}=\frac{2E_{rel0}}{k_{i}}$$

(14) $$\mathrm{and}\;{\Delta Q}_{1}{}^{2}=\frac{2E_{\mathrm{rel}1}}{k_{i}}$$

where $E_{rel0}$ and $E_{rel1}$ are the relaxation energy of the 0-0 and 0-1 transitions, respectively and $k_{i}$ is the force constant and it is equal to 490 N/m [59] for the C-H bond. Therefore, $E_{rel0}$ and $E_{rel1}$ can be calculated by [58]:

(15) $$E_{rel0}=\hbar \omega_{i}\;S_{0}$$

(16) $$E_{rel1}=\hbar \omega_{i}\;S_{1}$$

Moreover, the reduced ionic mass $M_{0}$ and $M_{1}$ values of the 0-0 and 0-1 transitions respectively can be deduced using Equation (11).

**Figure 9:**
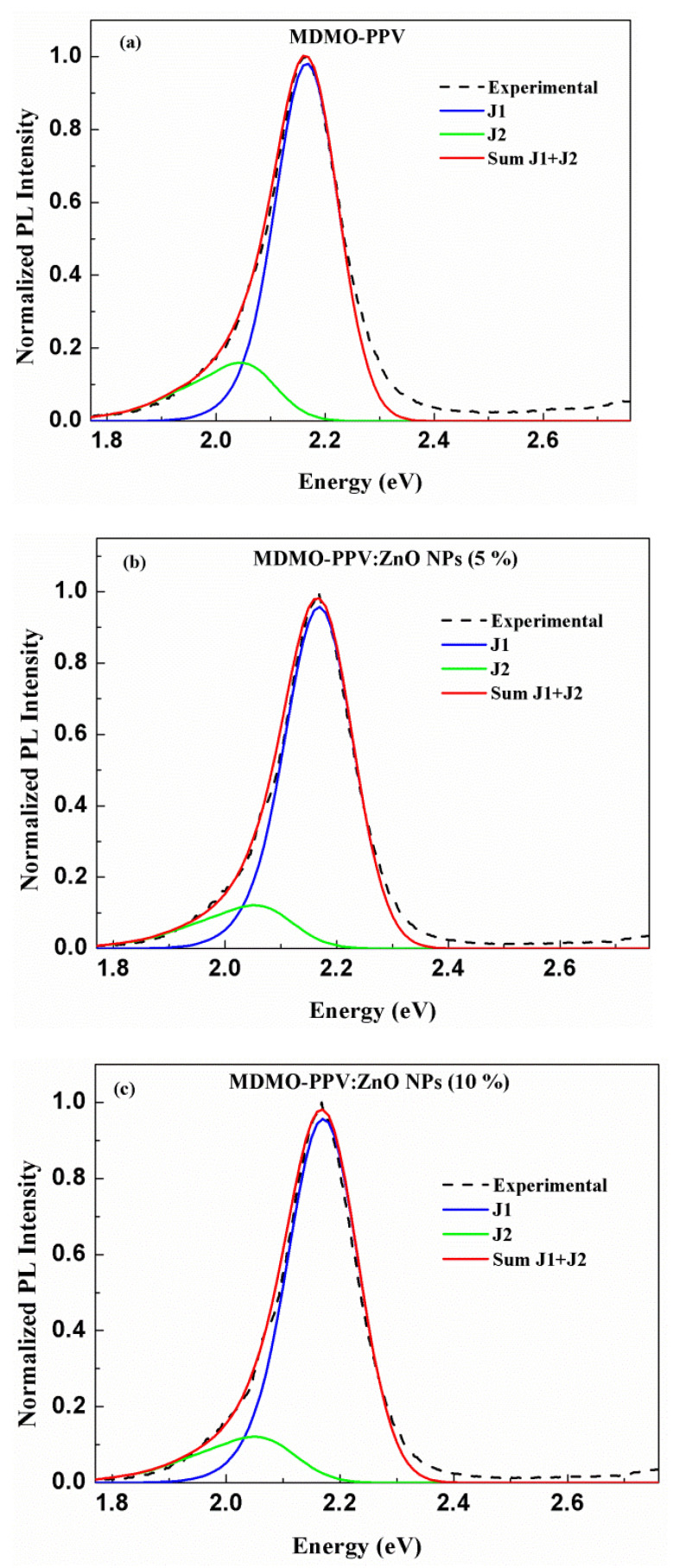
PL Experimental spectra (black dashed lines) and theoretical spectrum (red solid lines) (**a**) Pristine MDMO-PPV (**b**) MDMO-PPV: ZnO NPs (5%) (**c**) MDMO-PPV: ZnO NPs (10%); using the sum of FC progressions corresponding to emission from J-type aggregate species J_1_ (blue solid lines), and J_2_ (green solid lines).

The following equation can be used to model PL spectra [65]:

(17) $$I\left(\hbar \omega \right)\propto {\left(\hbar \omega \right)}^{3}e^{-S_{i}}\sum_{\nu_{i}}\frac{S_{i}^{\nu_{i}}}{\nu_{i}!}\Gamma \left(\hbar \omega -E_{0}+\nu_{i}\hbar \omega_{i}\right)$$

where Γ is the Gaussian function with constant width σ, and $E_{0}$ is the 0-0 transition energy. Figure 9 represents the experimental data of energy best fitting related to the normalized PL spectra of the MDMO-PPV pristine polymer and MDMO-PPV: ZnO NPs blends (5% and 10%). The involved mode 0.11 eV is detected by Raman spectroscopy and attributed to out-of-plane C-H stretching vibration of the vinylene group [67]. The modeling for the normalized PL spectra of the pristine polymer and the two blends was obtained by the total of the two intrachain FC progressions corresponding to emission from J-type aggregate species labeled J_1_ and J_2_. Also, in Figure 9 the black dashed lines are the experimental emission spectra, and the red lines are the theoretical spectra, for these samples. To perform the calculation and the PL spectrum of the polymer, we have used a sum of J-type aggregate species where J_1_ (blue solid lines) related to the transition from the lowest-excited singlet state to the lowest vibrational-level of the ground state (0–0 transition) of aggregated chains, and J_2_ (green solid line) related to a transition from the lowest-excited singlet state to the first-excited vibrational level of the ground state (0–1 transition) of aggregated chains. The PL spectra of blends of thin films are also modeled by the sum of aggregate species of the J type. The fitting parameters that have been calculated are presented in Table 3.

Table 3 shows the $E_{0-0}$ transition energy is centered at 2.163 eV for the pristine polymer, showing a blue shift upon ZnO NPs addition. Also, the peak position of the 0-1 transition is blue-shifted compared to the 0-0 transition of the MDMO-PPV thin film. These observed PL peak shifts are attributed to the alteration in the conjugation length of MDMO PPV chains [68]. Furthermore, the Huang-Rhys factor $S_{0}$, $S_{1}$ of each J_1_, and J_2_ aggregate of pristine polymer are increased with the increment of the nanoparticles’ concentration. S corresponds to conformational disorder [69]. The highest Huang-Rhys factor is obtained in films with greater conformational disorder. Conformational disorder reduces the polymer’s conjugation length, which increases the S factor [70]. This increase indicates that the inclusion of ZnO NPs in a sample of the polymer enhances the conformational disorder. MDMO-PPV: ZnO NPs blended at 5% and 10% reduce conjugation length and cause more conformational disorder than MDMO-PPV in its pristine form. The main reason for the increase in the S factor is the aggregation of the nanocomposite films with more ZnO NPs, confirmed by SEM images. As we mentioned previously, the intensity of the 0-1 transition increases for higher concentrations of ZnO NPs (see Figure 5), and the value of $S_{1}$ of the aggregate J_2_ (0-1 transition band) is varied from 0.548 to 0.600. Thus, this result indicates that the increase in the Huang-Rhys factor is related to more vibronic transitions.

Furthermore, the relaxation energy ($E_{rel}$) is enhanced in blended thin films compared to the pristine MDMO-PPV and reached 73.70 meV for 10% of the metal oxide and it is 66.33 meV in MDMO-PPV films. This increase is explained by the enhancement of the vibronic transitions. It is clear to chow from Table 3 that the emission spectrum of MDMO-PPV: ZnO NPs (10%) is broader where σ reached 0.061 eV and it is 0.057 eV for pristine polymer. The result obtained is due to the increase in the conformational disorder. The increase in conformational disorder results in a broader emission spectrum [71].

## 4. Conclusions

In summary, thin films based on MDMO-PPV polymer and ZnO blends in two amounts of 5% and 10% have been prepared. The incorporation of ZnO NPs increases the PL intensity of MDMO-PPV. This enhancement is due to the increase in the probability of exciton formation where more holes will recombine with trapped electrons in ZnO NPs. ZnO NPs increase the 0-1 transition band of the pristine polymer originating from J-aggregation. As a result of the presence of ZnO NPs in the polymer matrix, the intrachain interaction in the MDMO-PPV chain increases, and the emission of the J-aggregate types increases.

Moreover, the optical band gap energy of MDMO-PPV and MDMO-PPV: ZnO NPs blend thin films increased as ZnO NPs were added. This is due to the reduction of defects and localized states in the energy band gap of nanocomposites. The confirmation of this statement is supported by the computation of the Urbach energy, which is reduced by adding ZnO NPs as compared to the pristine MDMO-PPV. The Franck-Condon (FC) analysis of the PL spectra concludes that the blue-shifted transitions energy $E_{0-0}$, and $E_{0-1}\;$ of the polymer with respect to that of the two blends is related to the reduction of the conjugation length.

Furthermore, the increase in the S factor enhancement is related to the increase in conformational disorder. Greater conformational disorder exists in MDMO-PPV: ZnO NP thin films with 10% ZnO. Adding more ZnO NPs results in an enhancement of vibronic transitions due to an increase in the S factor.

## Figures and Tables

**Table 1:** The processing parameters of the polymeric solutions in 10 mL of toluene.

Active Layer	Zinc Oxide (mg)	Polymer (mg)	Toluene (mL)	Thickness (nm)
MDMO-PPV: ZnO NPs (0%)	0	40	10	45
MDMO-PPV: ZnO NPs (5%)	2	38	10	55
MDMO-PPV: ZnO NPs (10%)	5	36	10	90

**Table 2:** The Optical properties of Pristine MDMO-PPV and MDMO-PPV: ZnO NPs blended (5% and 10%) thin films.

Samples	$Low\;E_{g\;}$ (eV)	$High\;E_{g\;}$ (eV)	$E_{u}$ (eV)	n
MDMO-PPV	2.15	3.63	0.114	2.34
MDMO-PPV: ZnO NPs (5%)	2.06	3.82	0.091	2.31
MDMO-PPV: ZnO NPs (10%)	2.05	3.85	0.093	2.30

**Table 3:** Extracted parameters obtained from FC analysis of the PL spectra; the HR ($S_{i}$), the PL 0-0, and 0-1 transitions energy ($E_{0-i}$), the phonon energies of the involved mode ℏω, the width σ of the Gaussian functions Γ and the relaxation energy ($E_{rel}$).

Samples	$E_{0-0}$ (eV)	$E_{0-1}$ (eV)	$S_{0}$	$S_{1}$	$\omega_{i}$ × 10^16^ (s^−1^)	ℏω (eV)	$\;M_{i}$ × 10^−26^ (Kg)	$\Delta Q_{i}$ (eV.m. N^−1^)	$n_{f0-0}$ $n_{f0-1}$	σ (eV)	$E_{rel}$ (meV)
MDMO-PPV	2.163	2.050	0.055	0.548	0.016	0.11	1.9141 (S_0_) 1.9140 (S_1_)	0.0049 (S_0_) 0.0156 (S_1_)	11.974 (S_0_) 16.940 (S_1_)	0.057	66.33
MDMO-PPV: ZnO NPs (5%)	2.165	2.060	0.070	0.570	0.016	0.11	1.9141(S_0_) 1.9140 (S_1_)	0.0056 (S_0_) 0.0159 (S_1_)	12.038 (S_0_) 16.870 (S_1_)	0.060	70.40
MDMO-PPV: ZnO NPs (10%)	2.167	2.060	0.070	0.600	0.016	0.11	1.9141 (S_0_) 1.9140 (S_1_)	0.0056 (S_0_) 0.0164 (S_1_)	12.041 (S_0_) 16.750 (S_1_)	0.061	73.70

## Data Availability

Not applicable.

## References

[1] An Z., Huang Y., Zhang R. (2023). High-temperature multispectral stealth metastructure from the microwave-infrared compatible design. Compos. Part B Eng..

[2] An Z., Li Y., Luo X., Huang Y., Zhang R., Fang D. (2022). Multilaminate metastructure for high-temperature radar-infrared bi-stealth: Topological optimization and near-room-temperature synthesis. Matter.

[3] Komlev A.S., Gimaev R.R., Zverev V.I. (2021). Smart magnetocaloric coatings for implants: Controlled drug release for targeted delivery. Phys. Open.

[4] Liu G., Zhang X., Chen X., He Y., Cheng L., Huo M., Yin J., Hao F., Chen S., Wang P. (2021). Additive manufacturing of structural materials. Mater. Sci. Eng. R Rep..

[5] Yang Y., Song X., Li X., Chen Z., Zhou C., Zhou Q., Chen Y. (2018). Recent Progress in Biomimetic Additive Manufacturing Technology: From Materials to Functional Structures. Adv. Mater..

[6] Friend R.H., Gymer R., Holmes A., Burroughes J., Marks R., Taliani C., Bradley D., Santos D.D., Bredas J.-L., Lögdlund M. (1999). Electroluminescence in conjugated polymers. Nature.

[7] Jagadesan P., Yu Z., Barboza-Ramos I., Lara H.H., Vazquez-Munoz R., López-Ribot J.L., Schanze K.S. (2020). Light-activated antifungal properties of imidazolium-functionalized cationic conjugated polymers. Chem. Mater..

[8] Hestand N.J., Spano F.C. (2014). The effect of chain bending on the photophysical properties of conjugated polymers. J. Phys. Chem. B.

[9] Li X.-J., Sun G.-P., Gong Y.-F., Li Y.-F. (2023). Recent research progress of n-type conjugated polymer acceptors and all-polymer solar cells. Chin. J. Polym. Sci..

[10] Ture S.A., Pattathil S.D., Zing B.Z., Abbaraju V. (2023). Fluorescence Sensing of Some Important Nitroaromatic Compounds by Using Polyaniline Ag Composite. Micro.

[11] Desu M., Sharma S., Cheng K.-H., Wang Y.-H., Nagamatsu S., Chen J.-C., Pandey S.S. (2023). Controlling the molecular orientation of a novel diketopyrrolopyrrole-based organic conjugated polymer for enhancing the performance of organic field-effect transistors. Org. Electron..

[12] Alfadhli S., Darwish A., Soliman S., El-Zaidia E., Yahia I., Laariedh F., Alatawi A., Bahamran A., Alatawi N.M., Hamdalla T.A. (2023). Structural characterizations and photoelectric performance of non-crystalline boron subphthalocyanine chloride films/FTO for photodiode applications. J. Non-Cryst. Solids.

[13] Ibnaouf K. (2013). Excimer state of a conjugated polymer (MEH-PPV) in thin films. Opt. Laser Technol..

[14] Alsalhi M., Ibnaouf K., Masilamani V., Yassin O. (2007). Excimer state of a conjugate polymer (MEH-PPV) in liquid solutions. Laser Phys..

[15] Ibnaouf K., Prasad S., Masilamani V., AlSalhi M. (2013). Evidence for amplified spontaneous emission from double excimer of conjugated polymer (PDHF) in a liquid solution. Polymer.

[16] Prasad S., Ibnaouf K., AlSalhi M., Masilamani V. (2014). Laser from the dimer state of a conjugated polymer (PFO) in solution. Polymer.

[17] Rörich I., Schönbein A.-K., Mangalore D.K., Ribeiro A.H., Kasparek C., Bauer C., Crăciun N.I., Blom P.W., Ramanan C. (2018). Temperature dependence of the photo-and electroluminescence of poly (p-phenylene vinylene) based polymers. J. Mater. Chem. C.

[18] Bourbon S., Gao M., Kirstein S. (1999). Electroluminescence of self-assembled films of poly (p-phenylene vinylene) and J-aggregates. Synth. Met..

[19] Colaneri N., Bradley D., Friend R., Burn P., Holmes A., Spangler C. (1990). Photoexcited states in poly (p-phenylene vinylene): Comparison with trans, trans-distyrylbenzene, a model oligomer. Phys. Rev. B.

[20] Wu C., Chun J., Burrows P., Sturm J., Thompson M., Forrest S., Register R. (1995). Poly (p-phenylene vinylene)/tris (8-hydroxy) quinoline aluminum heterostructure light emitting diode. Appl. Phys. Lett..

[21] Masse M.A., Martin D.C., Thomas E.L., Karasz F.E., Petermann J.H. (1990). Crystal morphology in pristine and doped films of poly (p-phenylene vinylene). J. Mater. Sci..

[22] Marks R., Halls J., Bradley D., Friend R., Holmes A. (1994). The photovoltaic response in poly (p-phenylene vinylene) thin-film devices. J. Phys. Condens. Matter.

[23] Chasteen S.V., Sholin V., Carter S.A., Rumbles G. (2008). Towards optimization of device performance in conjugated polymer photovoltaics: Charge generation, transfer and transport in poly (p-phenylene-vinylene) polymer heterojunctions. Sol. Energy Mater. Sol. Cells.

[24] Oo T., Mathews N., Tam T., Xing G., Sum T., Sellinger A., Wong L., Mhaisalkar S. (2010). Investigation of photophysical, morphological and photovoltaic behavior of poly (p-phenylene vinylene) based polymer/oligomer blends. Thin Solid Film..

[25] Blayney A.J., Perepichka I.F., Wudl F., Perepichka D.F. (2014). Advances and Challenges in the Synthesis of Poly (p-phenylene vinylene)-Based Polymers. Isr. J. Chem..

[26] Al-Asbahi B.A., Alanezi A.A., AlSalhi M.S. (2022). Photophysical characteristics of multicolor emitting MDMO-PPV–DMP/ZnO hybrid nanocomposites. Molecules.

[27] Shin M.J., Gwon D.-O., Lee G.S., Ahn H.S., Yi S.N., Ha D.H. (2014). Fabrication of n-GaN/MDMO-PPV hybrid structures for optoelectronic devices. J. Lumin..

[28] Modwi A., Ali M., Taha K.K., Ibrahem M., El-Khair H., Eisa M., Elamin M., Aldaghri O., Alhathlool R., Ibnaouf K. (2018). Structural and optical characteristic of chalcone doped ZnO nanoparticles. J. Mater. Sci. Mater. Electron..

[29] Liao S.-H., Jhuo H.-J., Yeh P.-N., Cheng Y.-S., Li Y.-L., Lee Y.-H., Sharma S., Chen S.-A. (2014). Single junction inverted polymer solar cell reaching power conversion efficiency 10.31% by employing dual-doped zinc oxide nano-film as cathode interlayer. Sci. Rep..

[30] Dimitrov S.D., Schroeder B.C., Nielsen C.B., Bronstein H., Fei Z., McCulloch I., Heeney M., Durrant J.R. (2016). Singlet exciton lifetimes in conjugated polymer films for organic solar cells. Polymers.

[31] Ibnaouf K. (2020). Photodynamic properties of poly [2-methoxy-5-(3′, 7′-dimethyloctyloxy)-1, 4-phenylenevinylene] under pulsed laser excitation. Opt. Laser Technol..

[32] Quist P.A., Sweelssen J., Koetse M.M., Savenije T.J., Siebbeles L.D. (2007). Formation and decay of charge carriers in bulk heterojunctions of MDMO-PPV or P3HT with new n-type conjugated polymers. J. Phys. Chem. C.

[33] Rao M.M., Su Y.K., Huang T.-S., Tu M.-L., Wu S.-S., Huang C.-Y. (2010). Enhanced performance of polymer light emitting devices using zinc oxide nanoparticle with poly (vinylcarbazole). J. Electrochem. Soc..

[34] Musa I., Massuyeau F., Faulques E., Nguyen T.-P. (2012). Investigations of optical properties of MEH-PPV/ZnO nanocomposites by photoluminescence spectroscopy. Synth. Met..

[35] Lee C.-W., Renaud C., Hsu C.-S., Nguyen T.-P. (2008). Traps and performance of MEH-PPV/CdSe (ZnS) nanocomposite-based organic light-emitting diodes. Nanotechnology.

[36] Zhan X., Zhu D. (2010). Conjugated polymers for high-efficiency organic photovoltaics. Polym. Chem..

[37] Beek W.J., Wienk M.M., Janssen R.A. (2004). Efficient hybrid solar cells from zinc oxide nanoparticles and a conjugated polymer. Adv. Mater..

[38] Dhole S.G., Dake S.A., Prajapati T.A., Helambe S.N. (2018). Effect of ZnO filler on structural and optical properties of polyaniline-ZnO nanocomposites. Procedia Manuf..

[39] Cho M.S., Park S.Y., Hwang J.Y., Choi H.J. (2004). Synthesis and electrical properties of polymer composites with polyaniline nanoparticles. Mater. Sci. Eng. C.

[40] Aldaghri O.A., El-Badry B.A., Ali M.K.M., Ibnaouf K.H. (2022). Effect of Gamma Irradiation on the Optical Properties of the Conjugated Copolymer B-co-MP. Appl. Sci..

[41] Li J., Xie B., Xia K., Zhao C., Li Y., Hu S. (2018). Enhanced PL and EL properties of Alq_3_/nano-TiO_2_ with the modification of 8-vinyl POSS. Opt. Mater..

[42] Uthirakumar P., Lee Y.-S., Suh E.-K., Hong C.-H. (2008). Hybrid fluorescent polymer–zinc oxide nanoparticles: Improved efficiency for luminescence conversion LED. J. Lumin..

[43] Jarka P., Tański T., Matysiak W., Krzemiński Ł., Hajduk B., Bilewicz M. (2017). Manufacturing and investigation of surface morphology and optical properties of composite thin films reinforced by TiO_2_, Bi_2_O_3_ and SiO_2_ nanoparticles. Appl. Surf. Sci..

[44] Shanshool H.M., Yahaya M., Yunus W.M.M., Abdullah I.Y. (2016). Investigation of energy band gap in polymer/ZnO nanocomposites. J. Mater. Sci. Mater. Electron..

[45] El-Zahed H., El-Korashy A., Rahem M.A. (2002). Effect of heat treatment on some of the optical parameters of Cu_9_Ge_11_Te_80_ films. Vacuum.

[46] Urbach F. (1953). The long-wavelength edge of photographic sensitivity and of the electronic absorption of solids. Phys. Rev..

[47] Elliot S. (2005). The Physics and Chemistry of Solids.

[48] Tommalieh M., Zihlif A. (2010). Optical properties of polyimide/silica nanocomposite. Phys. B Condens. Matter.

[49] Mohammed M., Khafagy R., Hussien M.S., Sakr G., Ibrahim M.A., Yahia I., Zahran H. (2021). Enhancing the structural, optical, electrical, properties and photocatalytic applications of ZnO/PMMA nanocomposite membranes: Towards multifunctional membranes. J. Mater. Sci. Mater. Electron..

[50] Yahia I., Farag A., Cavas M., Yakuphanoglu F. (2013). Effects of stabilizer ratio on the optical constants and optical dispersion parameters of ZnO nano-fiber thin films. Superlattices Microstruct..

[51] Zyoud S.H., Almoadi A., AlAbdulaal T.H., Alqahtani M.S., Harraz F.A., Al-Assiri M.S., Yahia I.S., Zahran H.Y., Mohammed M.I., Abdel-wahab M.S. (2023). Structural, Optical, and Electrical Investigations of Nd2O3-Doped PVA/PVP Polymeric Composites for Electronic and Optoelectronic Applications. Polymers.

[52] Mohan S.R., Joshi M., Dhami T., Awasthi V., Shalu C., Singh B., Singh V. (2017). Charge transport in thin films of MDMO PPV dispersed with lead sulfide nanoparticles. Synth. Met..

[53] Raj Mohan S., Joshi M., Shalu C., Ghosh C., Mukharjee C., Kukreja L. (2015). Charge transport properties of MDMO PPV thin films cast in different solvents. J. Polym. Sci. Part B Polym. Phys..

[54] Ortiz-Morales A., Ortiz-Lopez J., Cruz-Zaragoza E., Gómez-Aguilar R. (2015). Thermoluminescence and photoluminescence analyses of MEH-PPV, MDMO-PPV and RU (bpy) 3 gamma-irradiated polymer thin films. Appl. Radiat. Isot..

[55] Samuel I., Rumbles G., Collison C., Friend R., Moratti S., Holmes A. (1997). Picosecond time-resolved photoluminescence of PPV derivatives. Synth. Met..

[56] Cuba M., Rathinavalli U., Thangaraju K., Muralidharan G. (2014). Synthesis and optical properties of ZnO incorporated Tris-(8-hydroxyquinoline) aluminum. J. Lumin..

[57] Uthirakumar P., Suh E.-K., Hong C.-H. (2008). Effect of zinc oxide incorporation on the morphology of tris (8-hydroxyquinoline) aluminum/zinc oxide hybrid nanomaterials. Thin Solid Film..

[58] Crupi I., Boscarino S., Strano V., Mirabella S., Simone F., Terrasi A. (2012). Optimization of ZnO: Al/Ag/ZnO: Al structures for ultra-thin high-performance transparent conductive electrodes. Thin Solid Film..

[59] He G., Li Y., Liu J., Yang Y. (2002). Enhanced electroluminescence using polystyrene as a matrix. Appl. Phys. Lett..

[60] Periyayya U., Kang J.H., Ryu J.H., Hong C.-H. (2011). Synthesis and improved luminescence properties of OLED/ZnO hybrid materials. Vacuum.

[61] Atta A., Abdel Reheem A., Abdeltwab E. (2020). Ion beam irradiation effects on surface morphology and optical properties of ZnO/PVA composites. Surf. Rev. Lett..

[62] Quan S., Teng F., Xu Z., Zhang T., Qian L., Liu D., Hou Y., Wang Y. (2006). Temperature effects on photoluminescence of poly [2-methoxy-5-(20-ethyl-hexyloxy)-1,4-phenylene vinylene]. Mater. Lett..

[63] Scharsich C., Fischer F.S., Wilma K., Hildner R., Ludwigs S., Köhler A. (2015). Revealing structure formation in PCPDTBT by optical spectroscopy. J. Polym. Sci. Part B Polym. Phys..

[64] Clark J., Silva C., Friend R.H., Spano F.C. (2007). Role of intermolecular coupling in the photophysics of disordered organic semiconductors: Aggregate emission in regioregular polythiophene. Phys. Rev. Lett..

[65] Saidani M., Benfredj A., Romdhane S., Kouki F., Bouchriha H. (2012). Role of intermolecular coupling and electron-nuclear coupling in the photophysics of oligothiophenes. Phys. Rev. B.

[66] Kanemoto K., Sudo T., Akai I., Hashimoto H., Karasawa T., Aso Y., Otsubo T. (2006). Intrachain photoluminescence properties of conjugated polymers as revealed by long oligothiophenes and polythiophenes diluted in an inactive solid matrix. Phys. Rev. B.

[67] Wise A.J., Precit M.R., Papp A.M., Grey J.K. (2011). Effect of Fullerene Intercalation on the Conformation and Packing of Poly-(2-methoxy-5-(3′-7′-dimethyloctyloxy)-1,4-phenylenevinylene). ACS Appl. Mater. Interfaces.

[68] Zou J., Le Rendu P., Musa I., Yang S.-H., Dan Y., That C.T., Nguyen T. (2011). Investigation of the optical properties of polyfluorene/ZnO nanocomposites. Thin Solid Film..

[69] Chang R., Hsu J., Fann W., Liang K., Chang C., Hayashi M., Yu J., Lin S., Chang E., Chuang K. (2000). Experimental and theoretical investigations of absorption and emission spectra of the light-emitting polymer MEH-PPV in solution. Chem. Phys. Lett..

[70] Lutich A.A., Jiang G., Susha A.S., Rogach A.L., Stefani F.D., Feldmann J. (2009). Energy transfer versus charge separation in type-ii hybrid organic–inorganic nanocomposites. Nano Lett..

[71] Quan S., Teng F., Xu Z., Qian L., Zhang T., Liu D., Hou Y., Wang Y., Xu X. (2007). Temperature dependence of photoluminescence in MEH-PPV blend films. J. Lumin..

